# Evaluation of a multiplex real-time PCR for detection of four bacterial agents commonly associated with bovine respiratory disease in bronchoalveolar lavage fluid

**DOI:** 10.1186/s12917-017-1141-1

**Published:** 2017-07-13

**Authors:** Henk J. Wisselink, Jan B.W.J. Cornelissen, Fimme J. van der Wal, Engbert A. Kooi, Miriam G. Koene, Alex Bossers, Bregtje Smid, Freddy M. de Bree, Adriaan F.G. Antonis

**Affiliations:** Wageningen Bioveterinary Research, P.O. Box 65, 8200 AB Lelystad, The Netherlands

**Keywords:** Bovine respiratory disease, PCR, *Histophilus somni*, *Mannheimia haemolytica*, *Pasteurella multocida*, *Trueperella pyogenes*

## Abstract

**Background:**

*Pasteurella multocida*, *Mannheimia haemolytica*, *Histophilus somni* and *Trueperella pyogenes* are four bacterial agents commonly associated with bovine respiratory disease (BRD). In this study a bacterial multiplex real-time PCR (the RespoCheck PCR) was evaluated for the detection in bronchoalveolar lavage fluid (BALF) of these four bacterial agents.

**Results:**

The analytical sensitivity of the multiplex real-time PCR assay determined on purified DNA and on bacterial cells of the four target pathogens was one to ten fg DNA/assay and 4 × 10^−1^ to 2 × 10^0^ CFU/assay. The analytical specificity of the test was, as evaluated on a collection of 118 bacterial isolates, 98.3% for *M. haemolytica* and 100% for the other three target bacteria. A set of 160 BALF samples of calves originating from ten different herds with health problems related to BRD was examined with bacteriological methods and with the RespoCheck PCR. Using bacteriological examination as the gold standard, the diagnostic sensitivities and specificities of the four bacterial agents were respectively between 0.72 and 1.00 and between 0.70 and 0.99. Kappa values for agreement between results of bacteriological examination and PCRs were low for *H. somni* (0.17), moderate for *P. multocida* (0.52) and *M. haemolytica* (0.57), and good for *T. pyogenes* (0.79). The low and moderate kappa values seemed to be related to limitations of the bacteriological examination, this was especially the case for *H. somni*.

**Conclusion:**

It was concluded that the RespoCheck PCR assay is a valuable diagnostic tool for the simultaneous detection of the four bacterial agents in BALF of calves.

## Background

In veal production, bovine respiratory disease (BRD) is the most common and economically important disease [1–3]. BRD is a multifactorial disease, involving multiple potentially pathogenic microorganisms that causes economic losses due to morbidity, mortality, medication costs, increased time on feeding and associated labour costs [4]. In the Dutch veal industry, calves at an age between ten days and five weeks are sold by dairy farmers to traders who transport calves to the calf collection centres, from where they are transported to fattening farms where they are mingled with other young calves, reared and fattened until slaughter at an age of 6–7 months. The young age of the calves, their inadequate immunity and several stress factors including transportation and mingling with other young calves makes that the calves are vulnerable for infections, a scenario which is recognised as resulting in the frequent occurrence of disease problems including BRD [5]. To protect calves, at the end of the twentieth century veal farmers commonly administered antibiotics prophylactically immediately after arrival on the farm. Growing concerns about the increase in antibiotic resistance including methicillin resistant *Staphylococcus aureus* (MRSA) and extended-spectrum βeta-lactamase producing *Escherichia coli* in livestock resulted in a policy aimed to substantially reduce the use of antibiotics in The Netherlands [6]. Part of this policy was a ban of all prophylactic use of antimicrobials in the Netherlands in 2012 (Besluit Diergeneesmiddelen, 2 November 2012; http://wetten.overheid.nl/BWBR0032386/2015-01-01). To implement and regulate this reduction in the use of antibiotics in the Netherlands, in recent years a collaboration was set up between stakeholders in livestock production and the national government [6, 7]. This lead to registration of all antibiotics administered to livestock at farm level [8] and to mandatory treatment and health plans to be developed for each farm to enforce that antimicrobial treatment is solely based on proper diagnosis [6]. However, the availability of rapid and reliable diagnostic tools is limited.

In BRD, several viral and bacterial pathogens may be involved [9–11]. For detection of these potential pathogens a variety of diagnostic tests have been described, including culture and molecular methods, see review by Fulton and Confer [12]. These methods all have their benefits and limitations in terms of speed of analysis, sensitivity and specificity [12]. In case of BRD, interpretation of results is sometimes challenging and diagnostic laboratories usually offer diagnostic tests for a limited number of potential pathogens which can be involved in BRD and which are sometimes also time-consuming. These are drawbacks that can hamper veterinarians and farmers for sending samples to diagnostic laboratory for analysis of BRD associated organisms. To offer veterinarians a fast and complete laboratory result, a set of three multiplex real-time PCRs was developed for detection of i) viral, ii) bacterial and iii) mycoplasma BRD-associated pathogens in bronchoalveolar lavage fluid (BALF) of calves, under the name RespoCheck (WBVR, Lelystad, The Netherlands). In this study the RespoCheck bacterial multiplex real-time PCR (abbreviated to RespoCheck PCR) was evaluated with commonly used culture methods as reference. This PCR has been developed for the detection of four bacterial pathogens commonly associated with BRD [9, 13], i.e. *Pasteurella multocida*, *Mannheimia haemolytica*, *Histophilus somni* and *Trueperella pyogenes*. The results showed that the RespoCheck PCR assay is a sensitive and valuable diagnostic tool suitable for the simultaneous detection of four BRD associated bacterial targets in BALF of calves.

## Methods

### Bacterial strains and growth conditions

A total of 118 bacterial isolates, representing 46 different species, were used for evaluation of specificity of the RespoCheck PCR. At least five representatives of each of the four target species, as well as bacterial strains from non-target species, consisting of isolates of phylogenetically closely related non-target species, and isolates of species from cattle with diseases other than BRD were included. Reference strains were obtained from the American Type Culture Collection (ATCC) and the Culture Collection of the University of Gothenburg (Sweden) (CCUB) and field strains were isolated from lungs of calves in a Dutch field study on BRD. An overview of the strains is provided in Table 1. Bacteria other than *H. somni* were grown overnight at 37 °C on heart infusion agar (ACU 7269C, Acumedia Manufacturers Inc. Lansing, MI) supplemented with 5% defibrinated sheep blood. *H. somni* was grown overnight at 37 °C in air with 5% CO_2_ on chocolate blood agar, using 7% defibrinated horse blood and Columbia blood agar (CM 331, Oxoid, Badhoevedorp, The Netherlands).

**Table 1:** List of 118 bacterial isolates and results of the RespoCheck bacterial multiplex real-time PCR

Identification^a^(number of isolates tested)	CCUG identification^b^	Source	RespoCheck bacterial multiplex real-time PCR(Ct values)			
			*P. multocida*	*M. haemolytica*	*H. somni*	*T. pyogenes*
*Acidovorax spp.* (*n* = 3)	NA^c^	WBVR collection^d^	- ^g^	-	-	-
*Actinomyces spp*	NA	WBVR collection^d^	-	-	-	-
*Aerococcus viridans*	NA	WBVR collection^e^	-	-	-	-
*Biberstenia trehalosi*	NA	WBVR collection^d^	-	-	-	-
*Brucella abortus*	NA	WBVR collection^e^	-	-	-	-
*Comamonas kerstersii*	NA	WBVR collection^d^	-	-	-	-
*Corynebacterium bovis* (*n* = 2*)*	NA	WBVR collection^e^	-	-	-	-
*Corynebacterium pseudotuberculosis*	NA	WBVR collection^e^	-	-	-	-
*Escherichia coli*	NA	WBVR collection^e^	-	-	-	-
*Gallibacterium anatis* (*n* = 5)	NA	WBVR collection^d^	-	-	-	-
*Hafnia alvei*	NA	WBVR collection^d^	-	-	-	-
*Histophilus somni*	NA	ATCC 22132^f^	-	-	15.2	-
*Histophilus somni* (*n* = 4)	NA	WBVR collection^d^	-	-	12.3–14.1	-
*Klebsiella oxytoca*	NA	WBVR collection^e^	-	-	-	-
*Klebsiella pneumoniae*	NA	WBVR collection^e^	-	-	-	-
*Lactobacillus mucosae*	NA	WBVR collection^d^	-	-	-	-
*Lactococcus garvieae*	NA	WBVR collection^e^	-	-	-	-
*Lactococcus lactis*	NA	WBVR collection^e^	-	-	-	-
*Listeria monocytogenes*	NA	WBVR collection^e^	-	-	-	-
*Mannheimia haemolytica*	NA	ATCC 14003^f^	-	16.7	-	-
*Mannheimia haemolytica* (*n* = 5)	NA	WBVR collection^d^	-	13.0–14.0	-	-
*Mannheimia haemolytica*	*Mannheimia ruminalis*	CCUG 38470-T	-	15.5	-	-
*Mannheimia haemolytica*	*Mannheimia glucosida*	CCUG 38457-T	-	16.0	-	-
*Mannheimia granulomatis*	*Mannheimia granulomatis*	CCUG 45422-T	-	-	-	-
*Mannheimia varigena*	*Mannheimia varigena*	CCUG 38462-T	-	-	-	-
*Micrococcus luteus*	NA	WBVR collection^e^	-	-	-	-
*Moraxella bovis*	NA	WBVR collection^e^	-	-	-	-
*Moraxelle lacunata* (*n* = 2)	NA	WBVR collection^d^	-	-	-	-
*Pantoea agglomerans* (*n* = 13)	NA	WBVR collection^d^	-	-	-	-
*Pasteurella multocida*	NA	ATCC 15743^f^	21.5	-	-	-
*Pasteurella multocida* (*n* = 23)	NA	WBVR collection^d^	15–17	-	-	-
*Pasteurella multocida*	Bisgaard Taxon 13	CCUG 16497	17.9	-	-	-
*Pasteurella multocida*	Bisgaard Taxon 13	CCUG 16498	17.6	-	-	-
*Pasteurella multocida*	*Pasteurella multocida* subsp. *gallicida*	CCUG 17978-T	18.0	-	-	-
*Pasteurella multocida*	*Pasteurella multocida* subsp. *septica*	CCUG 17977-T	17.0	-	-	-
Not typable	*Pasteurella aerogenes*	CCUG 27905	-	-	-	-
*Proteus mirabillis*	NA	WBVR collection^d^	-	-	-	-
*Pseudomonas aeruginosa*	NA	WBVR collection^e^	-	-	-	-
*Psychrobacter* spp.	NA	WBVR collection^d^	-	-	-	-
*Salmonella enterica* subsp. *enterica* serovar Dublin	NA	WBVR collection^e^	-	-	-	-
*Salmonella enterica* subsp. e*nterica* serovar Typhimurium	NA	WBVR collection^e^	-	-	-	-
*Serratia marcescans*	NA	WBVR collection^e^	-	-	-	-
*Staphylococcus aureus*	NA	WBVR collection^e^	-	-	-	-
*Staphylococcus epidermidis*	NA	WBVR collection^e^	-	-	-	-
*Streptococcus agalactiae*	NA	WBVR collection^e^	-	-	-	-
*Streptococcus bovis* (*n* = 5)	NA	WBVR collection^d^	-	-	-	-
*Streptococcus dysgalactiae*	NA	WBVR collection^e^	-	-	-	-
*Streptococcus faecalis*	NA	WBVR collection^e^	-	-	-	-
*Streptococcus pluranimalium* (*n* = 5)	NA	WBVR collection^d^	-	-	-	-
*Streptococcus pneumoniae*	NA	WBVR collection^e^	-	-	-	-
*Streptococcus* spp. (*n* = 3)	NA	WBVR collection^d^	-	-	-	-
*Streptococcus uberis*	NA	WBVR collection^e^	-	-	-	-
*Trueperella pyogenes*	NA	ATCC 9731^f^	-	-	-	15.8
*Trueperella pyogenes* (*n* = 5)	NA	WBVR collection^d^	-	-	-	12.8–16.0
*Yersinia enterolytica*	NA	WBVR collection^e^	-	-	-	-

^a^Isolates were identified with MALDI-TOF mass spectrometry (as described in Materials and Methods)

^b^CCUG: Culture Collection University of Gothenburg (Sweden)

^c^NA: Not applicable

^d^Isolated by Wageningen Bioveterinary Research (WBVR) from lungs of calves in the course of an earlier field study to BRD

^e^Cattle isolates from tissue other than lung

^f^ATCC: American Type Culture Collection (USA)

^g^-: Ct value above 40

### Field samples

In a Dutch field study on BRD from October 2013 till March 2014, veal calf farms (*n* = 10) were selected. On each farm, the study period started shortly after arrival of the calves (D0) and ended 84 days (D84) later. At the start (D0) and end (D84) of the study period and during outbreaks of BRD, bronchoalveolar lavage (BAL) samples were taken from calves without and with clinical problems of BRD (as defined by severe upper respiratory tract and/or lower respiratory tract disease).

BAL samples were collected as previously described [14]. Between 35 and 75 mL BAL was obtained from each calf after instillation of 100 mL PBS with 10% fetal calf serum. The BAL samples were transported within 24 h under cooled conditions (ice packs) to the laboratory. At the laboratory, clots of mucus were removed and 10 mL BAL was spun down for 10 min at 4600×g at 4 °C. The BAL pellet was used for direct bacteriological examination and the remaining BAL material was stored in 250 μL aliquots at −80 °C in the presence of 15% glycerol.

Bacteriological examination was performed using media and growth conditions as described above. Single colonies were subcultured twice and identified with MALDI-TOF mass spectrometry (Bruker MALDI Biotyper Microflex, version 3.1 with reference database V4.0 (5627 MSPs) Bruker Daltonics GmbH, Germany).

For evaluation of the RespoCheck PCR, BALF samples were selected from calves which were positive in bacteriological examination for one of the four BRD associated targets. In total 160 BALF samples were selected from the ten herds (with 12, 7, 7, 11, 9, 9, 5, 4, 6 and 90 samples being collected from these herds).

### RespoCheck PCR

Starting material for the DNA extraction was 200 μL of BAL pellet or a loopful of colonies from a pure culture on agar plates. DNA extraction was performed on the MagNA Pure LC Instrument (Roche Life Science) and carried out with the MagNA Pure LC Total NA Isolation Kit (Roche-Diagnostics, Almere, The Netherlands) according to the manufacturer’s instructions and using the “Total NA External lysis” protocol in the accompanying software (MagNA Pure LC Software Version 2.11) of the MagNA Pure LC Instrument.

The RespoCheck PCR reaction was on a 7500 Fast Real Time PCR system (Applied Biosystems, Bleiswijk, The Netherlands) using the QuantiFast Multiplex Kit RT-PCR kit (Qiagen, Venlo, The Netherlands).The PCR assay was run in a 20 μL reaction mix containing 5 μL of the nucleic acid sample, 250 nM of each primer, 100 nM of each MGB probe, 1 × QuantiFast Multiplex RT-PCR Master Mix (with ROX dye) and sterile deionised water. An initial denaturation/activation for 60 s at 95 °C was followed by 50 cycles of 10 s at 95 °C and 30 s at 60 °C.

The threshold cycle (Ct) value was determined for each sample by singleplex and multiplex real-time PCR with a threshold of 50% of the Delta Rn value (log). The threshold was manually set at 0.04 in the linear phase of the amplification plot, whereby the slope and correlation coefficient values were approximately 3.2 and 99.9% respectively. The PCR results were scored negative (−) when the generated Ct value was 40 or more and positive (+) for Ct values below 40.

A positive control of each of the four bacterial pathogens was included in every DNA isolation run and in each multiplex PCR reaction. Positive controls were prepared using the ATCC supplied strains of the four bacterial species (Table 1). *P. multocida*, *M. haemolytica* and *T. pyogenes* were cultured in brain heart infusion broth (BHI) broth (CM 225; Oxoid) and *H. somni* was cultured in BHI supplemented with 5 μg/mL β-nicotinamide adenine dinucleotide (NAD, Calbiochem, La Jolla, CA). To obtain early stationary-phase cultures, the overnight cultures were diluted (1:100) and incubated at 37 °C. Incubation was stopped after approximately 4 h at an optical density of 0.5 at 600 nm. Cultures containing approximately 1 × 10^9^ CFU/mL were then centrifuged at 4000 x g for 15 min. And stored at −80 °C in Nutrient broth No. 2 with 15% glycerol. In every DNA isolation run a “reagent blank” control (sterile water) was included and in each multiplex PCR run a “no-template” control (also sterile water).

### Sensitivity and specificity

The analytical sensitivity of the RespoCheck PCR was defined as the ability of the assay to detect the lowest concentration of DNA/assay and CFU/assay [15] of the four target bacteria in PBS and BALF. For determination of the analytical sensitivity on DNA, a mixture was prepared of 10 ng DNA per assay of each of the four target bacteria. Ten-fold serial dilutions (*n* = 7) of this mixture were prepared in PBS resulting in a range from 10 ng to 1 fg DNA per assay of each of the four target bacteria. The Ct was determined for each sample by the singleplex and multiplex real-time PCR for each of the four target bacteria. For determination of the analytical sensitivity on bacterial cells in BALF, a mixture of cells of the four target bacteria was prepared in BALF of specific pathogen free (SPF) calves of 3–4 weeks old. Ten-fold serial dilutions (*n* = 7) of this mixture were prepared in BALF and DNA was isolated from these mixtures as described above. This resulted in a range from a 1 × 10^6^ to 1 × 10^−1^ *P. multocida* CFU/assay, 6 × 10^5^ to 6 × 10^−2^ *M. haemolytica* CFU/assay, 4 × 10^6^ to 4 × 10^−1^ *H. somni* CFU/assay and 6 × 10^5^ to 6 × 10^−2^ *T. pyogenes* CFU/assay. The Ct was determined for each sample by the singleplex and multiplex real-time PCR for each of the four target bacteria.

The analytical specificity was defined as the ability of the assay to distinguish the target organisms from non-target organisms [15]. For determining the analytical specificity of the RespoCheck PCR a panel of 118 bacterial isolates was used (Table 1).

The diagnostic sensitivity was defined as the proportion of samples from known infected reference animals that tested positive in an assay and the diagnostic specificity as the proportion of samples from known uninfected reference animals that tested negative in an assay [15]. The diagnostic sensitivity and specificity with 95% confidence intervals (95% CI) of the RespoCheck PCR was calculated for each target species using the result of the bacteriological examination of the BALF samples of the calves as reference method. Cohen’s kappa coefficient with 95% CI was calculated to describe to what extent the RespoCheck PCR agrees with the bacteriological examination. For calculations the DAG_Stat spreadsheet was used [16]. Kappa values were interpreted as follows: Kappa = 0.00–0.20, poor agreement; Kappa = 0.21–0.40, fair agreement; Kappa = 0.41–0.60, moderate agreement; Kappa = 0.61–0.80, good agreement; Kappa = 0.81–1.00, near-perfect agreement [17].

## Results

### Analytical sensitivity and specificity

**Fig. 1:**
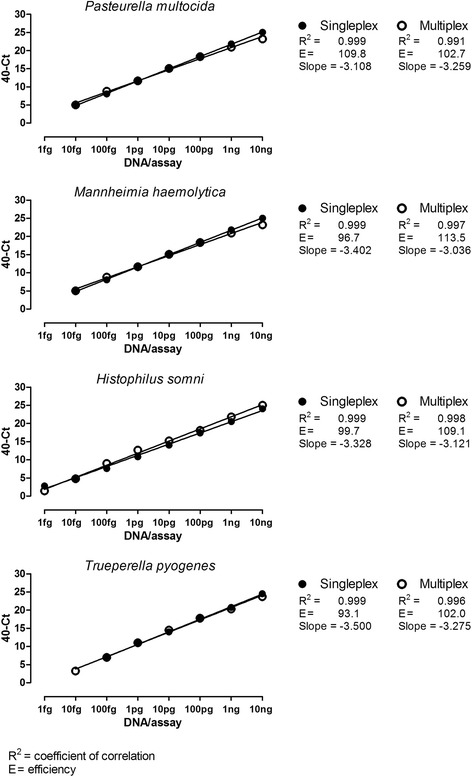
Analytical sensitivity of the RespoCheck bacterial real-time PCR determined in singleplex and multiplex assays on mixtures of DNA of *P. multocida*, *M. haemolytica*, *H. somni* and *T. pyogenes* isolates

The analytical sensitivity of the RespoCheck PCR was determined on mixtures of 10-fold serial dilutions of DNA of the four target bacteria and compared with the sensitivity of the singleplex PCRs. Positive multiplex real-time PCR results (Ct < 40) were obtained on at least one fg of chromosomal DNA of *H. somni* and ten fg chromosomal DNA of *P. multocida*, *M. haemolytica* and *T. pyogenes* (Fig. 1). Compared to the multiplex real-time PCR, the singleplex real-time PCR for *T. pyogenes* was less sensitive (one step in the serial dilution) (Fig. 1). For the other three target bacteria the sensitivity of the singleplex was similar to the multiplex real-time PCR (Fig. 1). The coefficient of correlation (R^2^) between the Ct values and the amount of DNA/assay was higher than 0.99 for all assays. The slopes varied between 3.036 (multiplex real-time PCR for *M. haemolytica*) and 3.500 (singleplex real-time PCR for *T. pyogenes*) which corresponds to an efficiency (E) of 113.5% and 93.1% respectively (Fig. 1).

**Fig. 2:**
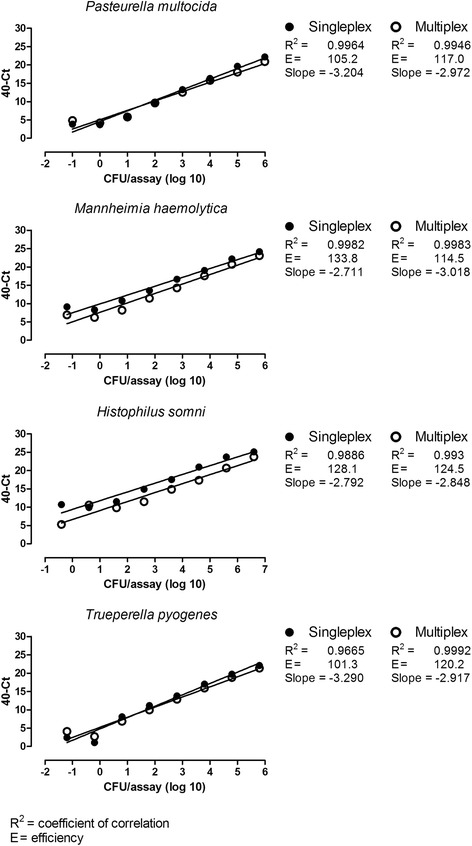
Sensitivity of the RespoCheck bacterial real-time PCR determined in singleplex and multiplex assays on BALF spiked with mixtures of cells of *P. multocida*, *M. haemolytica*, *H. somni* and *T. pyogenes* isolates

The analytical sensitivity of the RespoCheck bacterial multiplex real-time PCR was also determined in BALF of SPF calves, spiked with ten-fold serial dilutions of mixtures of cells of the four target bacteria (Fig. 2). Positive PCR results (Ct < 40) were obtained from the highest dilution corresponding to 1 × 10^−1^ *P. multocida* CFU/assay, 6 × 10^−2^ *M. haemolytica* CFU/assay, 4 × 10^−1^ *H. somni* CFU/assay and 6 × 10^−2^ *T. pyogenes* CFU/assay, both in single- and in multiplex real-time PCRs (Fig. 2). A good linear correlation (R^2^ > 0.96) between the Ct values and the number of CFU/assay was found for all assays. The slopes ranged from 2.711 (singleplex real-time PCR for *M. haemolytica*) to 3.290 (singleplex real-time PCR for *T. pyogenes*) which corresponds to an efficiency (E) of 133.8% and 101.3% respectively (Fig. 1).

The RespoCheck PCR was evaluated on a panel of 118 isolates including the four target species (Table 1). The results of the RespoCheck PCR showed that for isolates of the four target species Ct-values were obtained below 40 (Table 1). On the reference strains of *M. ruminalis* and *M. glucosida* also positive test results were obtained in the *M. haemolytica* PCR, indicating a cross reaction. The other bacteria tested all negative (Table 1).

### Diagnostic sensitivity and specificity

A set of 160 BALF samples of calves, originating from herds with health problems related to BRD, was examined using bacteriological culture as well as the RespoCheck PCR. Diagnostic sensitivity and specificity, and Cohen’s Kappa Coefficient for the four target species in the multiplex real-time PCR, were calculated with the results of the bacteriological culture as reference. For three of the four target pathogens, a high number of the RespoCheck PCR-positive samples tested bacteriologically positive; 53 of 82 PCR-positives for *P. multocida*, 46 of 76 for *M. haemolytica* and 21 of 22 for *T. pyogenes*). A low number of RespoCheck PCR -positive samples tested bacteriologically positive for *H. somni* (5 of 42). For all four target pathogens, a high number of RespoCheck PCR -negative samples tested bacteriologically negative (68 of 78 for *P. multocida*, 80 of 84 for *M. haemolytica*, 118 of 118 for *H. somni* and 125 of 133 for *T. pyogenes*) (Table 2), resulting in moderate to high diagnostic sensitivities (resp. 0.84, 0.92, 1.00, and 0.72) and in moderate to high diagnostic specificities (resp. 0.70, 0.73, 0.76, 0.99) (Table 2). Kappa values for agreement between results of bacteriological examination and RespoCheck PCRs were low for *H. somni* (0.17), moderate for *P. multocida* (0.52) and *M. haemolytica* (0.57), and good for *T. pyogenes* (0.79) (Table 2).

**Table 2:** Results of bacteriological examination (BE) and of the RespoCheck bacterial multiplex real-time PCR of BALF samples of calves originating from herds with BRD associated health problems

Agent	No (%) of BALF samples					Diagnostic Sensitivity	Diagnostic Specificity	Cohen’s Kappa Coefficient
	BE + PCR +	BE + PCR-	BE ­ PCR +	BE - PCR -	Total			
*P. multocida*	53 (33)	10 (6)	29 (18)	68 (43)	160	0.84 (0.73–0.92)^a^	0.70 (0.60–0.79)	0.52 (0.39–0.64)
*M. haemolytica*	46 (28)	4 (3)	30 (19)	80 (50)	160	0.92 (0.81–0.98)	0.73 (0.63–0.81)	0.57 (0.45–0.69)
*H. somni*	5 (3)	0 (0)	37 (23)	118 (74)	160	1.00 (NA)^b^	0.76 (0.69–0.83)	0.17 (0.04–0.30)
*T. pyogenes*	21 (13)	8 (5)	1 (1)	125 (81)	155	0.72 (0.53–0.87)	0.99 (0.96–0.99)	0.79 (0.65–0.92)

^a^95% CI

^b^NA: Not applicable

To corroborate the results of the bacteriological examination for *H. somni*, ten BALF samples that were bacteriologically negative and PCR-positive for *H. somni* were cultured again for *H. somni*. No *H. somni* bacteria were detected in these ten samples.

## Discussion

To improve the detection and identification of BRD-associated pathogens in BALF samples, real-time PCR tests were developed under the name RespoCheck (WBVR, Lelystad, The Netherlands). In this study, the analytical and diagnostic sensitivity and specificity was evaluated of the RespoCheck PCR targeting four bacterial agents associated with BRD, i.e. *P. multocida*, *M. haemolytica*, *H. somni* and *T. pyogenes*. The results showed that the RespoCheck PCR assay is specific and sensitive for the detection of these four bacterial agents. The RespoCheck PCR is easy to perform and allows large-scale application as 96 samples can be run simultaneously. Compared to standard bacteriological assays, the RespoCheck PCR is much more rapid to perform. Therefore, this RespoCheck PCR may be an important diagnostic tool for bacterial pathogens associated with BRD in calves. The results can be used by veterinarians and farmers for selection of specific intervention measures, including appropriate biosecurity, vaccination and possible antimicrobial treatments [12, 18].

Recently, we evaluated a triplex real-time PCR for detection of in BALF of *Mycoplasma (M.) dispar*, *M. bovis* and *M. bovirhinis* [19]. These three Mycoplasma’s are associated with BRD [20] and application of the multiplex bacterial real-time PCR and the triplex Mycoplasma real-time PCR together improves detection of BRD-associated pathogens further.

In terms of labour and costs, a multiplex PCR approach is more favourable approach than several singleplex assays run in parallel. However, in a multiplex assay the sensitivity of the test can be affected for example due to competition for the reagents in the assay. Therefore we determined the analytical sensitivity in singleplex and in multiplex real-time PCR assays. The results showed that the analytical sensitivity was equally high for the singleplex and multiplex real-time PCR (Figs. 1 and 2) demonstrating that the sensitivity of the individual PCRs was not reduced when multiplexing the PCR assays.

The RespoCheck PCR showed a high analytical sensitivity as the number of CFU/assay detected ranged from 0.6 × 10^−1^ to 4 × 10^−1^. Primers and probes of the RespoCheck PCR are based on the V3 region of the 16S rDNA of the four bacterial target pathogens. Bacterial cells can contain multiple copies of the 16S rDNA gene [21] and this number influences the analytical sensitivity of the multiplex real-time PCR. *P. multocida*, *M. haemolytica*, *H. somni* and *T. pyogenes* contain six, six, five and two 16S rDNA copies per cell respectively (https://rrndb.umms.med.umich.edu). The analytical sensitivity is for *P. multocida*, *M. haemolytica*, *H. somni* and *T. pyogenes* is, in theory, 6 × 10^−1^, 4 × 10^−1^, 2 × 10^0^ and 1 × 10^−1^ CFU/assay respectively. In summary, the RespoCheck PCR can detect 4 × 10^−1^ to 2 × 10^0^ CFU/assay of each of the four target pathogens. The sensitivity as determined on purified DNA, was found to be one to ten fg DNA/assay and assuming 4.6 fg of DNA per bacterial cell [22] the sensitivity is 2 × 10^−1^ to 2 × 10^0^ CFU/assay for the RespoCheck PCR. It seems that sensitivity of the RespoCheck PCR as determined on DNA and on bacterial cells in BALF is similar. Apparently, BALF does not influence the analytical sensitivity of the RespoCheck PCR.

In the RespoCheck PCR, the reference strains of the four target bacteria resulted in positive PCR results (Ct-values <40) (Table 1). Two reclassified *P. multocida* isolates, Bisgaard Taxon 13 strains CCUG 16497 and CCUG 16498, were correctly identified as *P. multocida* in the multiplex real-time PCR (Table 1). As these strains originally were classified as *P. avium* biovar 2 [23], these results underline the high analytical specificity of the RespoCheck PCR.

For determining the analytical specificity, 118 bacterial isolates representing 46 different species including the target species were examined in the RespoCheck PCR (Table 1). This panel consisted of strains that were isolated in the course of an earlier study to BRD in calves (Table 1; Source WBVR collection^d^) including the target species but also nontarget species. For *P. multocida*, *H. somni* and *T. pyogenes* only the target species were positive in the RespoCheck PCR, and these results confirm the analytical specificity of the RespoCheck PCR. The reference strains for *M. glucosida* and *M. ruminalis* were positive for *M. haemolytica* in the RespoCheck PCR whereas negative results were expected according to the CCUG identification (Table 1) and the phylogenetic analysis by 165 rRNA comparison of these strains [24]. Interestingly, MALDI-TOF MS identified *M. ruminalis* as “probable” an *M. haemolytica* (score 1.789) and *M. glucosida* as “highly probable” an *M. haemolytica* (score 2.257). Thus the RespoCheck PCR results for *M. haemolytica* seemed to be in agreement with the MALDI-TOF MS results. An explanation could be that in the reference database of the Bruker MALDI Biotyper (V4.0) there are no *M. ruminalis* isolates and only one *M. glucosidase* isolate (DSM 19638 T). The results by MALDI-TOF MS suggest that there is a lack of differentiation using this method between *M. haemolytica*, *M. ruminalis* and *M. glucosidase*. This lack of differentiation was also observed using 16S rRNA gene sequencing and a tRNA-intergenic spacer PCR [24, 25]. On the other hand, a clear separation between the species of the genus *Mannheimia* is possible by real-time PCR or multiplex PCR on other targets [26, 27]. The consequences of the lack in differentiation for interpretation of the results of the RespoCheck PCR between these *Mannheimia* strains seems to be limited since *M. ruminalis* and *M. glucosida* are mainly associated with sheep [24].

Comparison of the results of the RespoCheck PCR with bacterial culture on BALF samples showed that the PCR assay was more frequently positive than the bacteriological examination. Similar observations concerning differences between PCR and culture of these pathogens in lung specimens have been reported by others [9, 28]. Low levels of bacterial cells (live or dead) and overgrowth by contaminating microflora in the BALF specimens may explain the differences in results obtained with the two methods. Suboptimal transport conditions or presence of antibiotics could also influence the viability of the bacterial cells, both, as previously suggested [29] leading to false negative results.

The difference in number of *H. somni* culture positive samples and *H. somni* RespoCheck PCR positive samples highlights the difficulties in isolating this organism. In other studies on *H. somni*, differences were found between PCR and culture of lung specimens [28–30]. The explanation for this difference is that *H. somni* is a slow growing organism with small colonies that can be easily overgrown by other organisms [30]. These findings explain also the low kappa values for agreement between results of bacteriological examination and multiplex real-time PCRs for *H. somni* (0.17). This low kappa value seems relate to a low sensitivity of the bacteriological examination of BALF for *H. somni* and for this reason the use of the multiplex real-time PCR may be preferable over bacteriological examination.

The evaluation of the RespoCheck PCR on BALF samples from naturally infected calves showed that, for *P. multocida*, *M. haemolytica* and *T. pyogenes*, there were a number of samples (respectively 10, 4 and 8) which were positive by cultivation and negative by RespoCheck PCR. Analysis of these results showed that the majority of these culture positive samples was on the basis of the presence of one single colony only (results not shown). Thus although PCR on BALF samples is far more sensitive than bacteriological examination (as described above), the PCR can also be false negative.

## Conclusion

The RespoCheck bacterial multiplex real-time PCR-test has been shown to represent a sensitive and reliable test for the simultaneous detection of *P. multocida*, *M. haemolytica*, *H. somni* and *T. pyogenes* in BALF samples of calves.

## Abbreviations

ATCC: American Type Culture Collection
BALF: Bronchoalveolar lavage fluid
BRD: Bovine respiratory disease
CCUB: Culture Collection of the University of Gothenburg (Sweden)
PCR/DGGE: PCR with denaturing gradient gel electrophoresis fingerprinting
